# A new qualitative RT-PCR assay detecting SARS-CoV-2

**DOI:** 10.1038/s41598-021-98114-5

**Published:** 2021-09-23

**Authors:** Marco Favaro, Walter Mattina, Enrico Salvatore Pistoia, Roberta Gaziano, Paolo Di Francesco, Simon Middleton, Silvia D’Angelo, Tullio Altarozzi, Carla Fontana

**Affiliations:** 1 Department of Experimental Medicine, “Tor Vergata” University, Via Montpellier 1, 00133 Rome, Italy; 2 Laboratory of Microbiology, Microbiology and Virology Lab, Tor Vergata University Hospital, V.le Oxford 81, 00133 Rome, Italy; 3 LifeGene Srl Messina, Italy Via Garibaldi 377, 98121 Messina, Italy; 4 Adaltis R&D S.R.L., Via Luigi Einaudi 7, 00012 Guidonia Montecelio, Italy

**Keywords:** Virology, Biotechnology, Microbiology

## Abstract

The world is facing an exceptional pandemic caused by SARS-CoV-2. To allow the diagnosis of COVID-19 infections, several assays based on the real-time PCR technique have been proposed. The requests for diagnosis are such that it was immediately clear that the choice of the most suitable method for each microbiology laboratory had to be based, on the one hand, on the availability of materials, and on the other hand, on the personnel and training priorities for this activity. Unfortunately, due to high demand, the shortage of commercial diagnostic kits has also become a major problem. To overcome these critical issues, we have developed a new qualitative RT-PCR probe. Our system detects three genes—RNA-dependent RNA polymerase (RdRp), envelope (E) and nucleocapsid (N)—and uses the β-actin gene as an endogenous internal control. The results from our assay are in complete agreement with the results obtained using a commercially available kit, except for two samples that did not pass the endogenous internal control. The coincidence rate was 0.96. The LoD of our assay was 140 cp/reaction for N and 14 cp/reaction for RdRp and E. Our kit was designed to be open, either for the nucleic acid extraction step or for the RT-PCR assay, and to be carried out on several instruments. Therefore, it is free from the industrial production logics of closed systems, and conversely, it is hypothetically available for distribution in large quantities to any microbiological laboratory. The kit is currently distributed worldwide (called MOLgen-COVID-19; Adaltis). A new version of the kit for detecting the S gene is also available.

## Introduction

Since November 2019, the world has had to deal with an unprecedented public health emergency: the coronavirus SARS-CoV-2 and the disease COVID-19^1–3^. The pandemic has put pressure on the health systems worldwide and has placed serious diagnostic difficulties on microbiologists who are called upon to respond to medical needs without valid scientific evidence, especially in the early stages^4–6^. Over time and through the gradual acquisition of scientific findings, supported by the World Health Organization (WHO), the Centers for Disease Control (CDC) and the European Centers for Disease Control (ECDC), evidence about the virus has become increasingly available. Access to knowledge has made it possible to design diagnostic kits for the detection of viruses in biological samples. Many diagnostic systems have been proposed over the months that often differ in genetic targets^7–11^.

Gradually, the diagnostic systems have passed the appropriate validations required by the Food and Drug Administration (FDA) or CE IVD. However, the massive global spread of the virus has caused difficulties in the steady supply of diagnostic systems on the market; sometimes, there is a shortage of only viral nucleic acid extraction systems, while other times, the shortage is in amplification and detection systems, and frequently both^7^. The shortage of diagnostic systems has imposed a consequent limitation on their use, which means that fewer test kits are available, and this causes delays in the identification of positive patients^12–14^. Unfortunately, to date, even in Italy, the problem still persists due to important differences between regions. On the one hand, we have to attend to the legitimate request of the population to access testing; on the other hand, we have to face the same request from health institutions and make use of the technological and diagnostic resources available. Another key element is the shortage of human resources engaged in microbiology laboratories to process COVID-19 tests.

After the maximum spread of the virus was reached, the real challenges for Italy and the rest of the world have turned to the storage of diagnostic tests, the simplicity in the administration of tests, and the sustainability of the entire system. This latter aspect is particularly relevant, especially now that we are on the threshold of a second wave of infections^15^.

At a time of great diagnostic difficulty, our research team designed a new diagnostic system that can not only meet the sensitivity and specificity requirements but can also provide a reasonably fast diagnosis. This means that we are introducing a faster diagnostic process at the height of the requests from health care systems. Our assay (hereafter, kit) is a probe-based qualitative reverse transcriptase real-time PCR (qRT–PCR) probe that detects COVID-19 target genes. In this paper, we present the characteristics of our system.

## Materials and methods

### Specimens for the initial assay evaluation

The remaining specimens collected during routine clinical care (166 nasopharyngeal swabs), which would otherwise have been discarded, were used in the evaluation of our system. Samples were collected by trained personnel using nasopharyngeal Eswab (Copan, Brescia-Italy) and processed at LifeGene srl Laboratory Messina (Italy) using a commercially available system: Novel Coronavirus Real-Time Multiplex RT-PCR Kit (2019-nCoV) (3-gene detection) (Life River, San Diego, CA, USA). The Life River system is one of those included in the list approved by the WHO^16,17^.

### Specimens for comparison with other commercial kits

To evaluate the performance of our assay, we tested it head to head with three other commercially available kits, namely, the RIDAGENE SARS-CoV-2 test (R-Biopharm AG, Darmstadt, Germany), which is a single target gene assay (detecting the E gene); the Real-Time Fluorescent RT-PCR Kit for Detecting SARS-CoV-2, which identifies the ORF1ab gene as a domain target (BGI Genomics Co. Ltd. Yantia, China); and the Allplex SARS-CoV-2 assay, which detects four target genes: the RdRp/S and N genes specific for SARS-CoV-2 and the E gene for all *sarbecoviruses,* including SARS-CoV-2 (Seegene Inc., Seoul, South Korea). A total of 40 remnants of specimens collected for routine clinical care (40 nasopharyngeal swabs) were used for the test comparison.

### Kit design

Our diagnostic assay is a probe-based qualitative reverse transcriptase real-time PCR (qRT-PCR) probe. The targeted COVID-19 genes detected by our assay are the RNA-dependent RNA polymerase (RdRp), envelope (E), and nucleocapsid (N) of COVID-19. The primers and probes were designed based on the published sequence of COVID-19 in NCBI (reference sequence NC 045512.2) and were synthesized by Bio-Fab Research (Bio-Fab Research, Rome, Italy). The two sets of primers were specific to COVID-19: the “E” primer for the envelope gene and the “N” primer for the nucleocapsid gene. One of them is called RdRp, which targets the polymerase gene and is common with the SARS virus. The concentrations of primers and probes were determined by experimental procedures, and the sensitivity of the test was carried out with the chimeric plasmid described below. Primer and probe sequences are not shown as the kit is protected intellectual property (MOLgen-COVID-19; SARS-CoV-2 Real Time RT-PCR kit, by Adaltis).

A portion of an endogenous human β-actin gene was used as an internal control (IC) for the test, the latter also allowing the evaluation of correct nasopharyngeal sampling.

To evaluate our kit, an initial proficiency assay was carried out in the microbiology laboratories of the Department of Experimental Medicine of the “Tor Vergata” University of Rome. This proficiency assay was run using chimeric plasmids (CPs) in which virus sequences were artificially inserted into a plasmid [pBlueScript II SK(+)]. The synthesis of CP was contracted for Bio-Fab Research (Bio-Fab Research, Rome, Italy).

The analytical specificity and cross-reactivity of the primers and probes in our kit were evaluated using ZeptoMetrix panels (ZeptoMetrix, Co., Buffalo, NY, USA): (a) NATtrol SARS-CoV-2 (E/ORF/1ab recombinant) Stock (ZeptoMetric); (b) NATtrol SARS-CoV-2 (recombinant-only N) Stock; and (c) NATtrol Coronavirus-SARS Stock. Stock is formulated with intact and purified bacterial cells that contain synthetic SARS-CoV-2 sequences (the cells have been chemically modified to render them noninfectious and stable in a refrigerator) (Table 4). The panels are supplied in a purified protein matrix that mimics the composition of real clinical samples. Cross-reactivity was evaluated using (1) the NATtrol Respiratory Verification Panel (Zeptometrix NATRVP2-BIO) containing 22 viral and bacterial targets and (2) NATtrol MERS-CoV Stock (NATMERS-ST). Both panels contained intact viral and/or bacterial particles chemically modified to render them noninfectious and stable in a refrigerator.

The analytical sensitivity or limit of detection (LoD) of the test was determined by serial dilution with the AccuPlex SARS-CoV-2 v2 Reference Material Kit containing 5175 RNA cp/mL of inactivated SARS-CoV-2 virus and the research reagent for SARS-CoV-2 RNA (NIBSC code 19/304) obtained from the National Institute for Biological Standards and Control (NIBSC, UK). We treated the samples from both panels as a common nasopharyngeal sample but in triplicate (Table 5).

### Assay conditions

A 200-µl aliquot of samples collected in Eswab was extracted with a manual procedure using the magnetic silica bead procedure (MOLgen Universal Extraction Kit, QIAamp viral RNA) according to the manufacturer’s instructions. To process many samples at once, the extraction procedure was also automated at ExtraLab (Adalties Srl, Guidonia, Italy).

Real-time amplification was performed with AmpliLab (Adalties Srl) and CFX96 (Bio-Rad, Hercules, CA, USA) using qPCRBIO PROBE 1-Step Go No-Rox (PCR biosystems; www.pcrbio.com). To establish the appropriate amount of reverse transcriptase activity, RTase Go quantitation was performed according to the manufacturer’s instructions. The titration experiment showed that 0.2 µl of 20× RTase Go in the amplification mix gave good results in terms of sensitivity.

We achieved the ideal reaction condition using a 20-µl reaction master mix composed as follows: 2× qPCRBIO probe 1 step Go Mix 10 µl; ppMix (a mixture of primer and probe) 5 µl; 20X RTase Go 0.2 µl; and 5 µl of sample. In particular, ppMix contained the following final concentrations: 10 pmol of RdRp, E and β-actin (forward and reverse primers), 30 pmol of N (forward and reverse primer), and 2.5 pmol of the probe for each target.

The RT-PCR conditions for both instruments were as follows: one step at 45 °C for 10 min; a step at 95 °C for 2 min; 40 steps at 95 °C for 5 s; and the last step at 60 °C for 25 s.

The instrument was programmed to read the RdRp gene in Fam, the E gene in Rox, the N gene in Cy5, and β-actin in the Hex channel.

The kit is now distributed worldwide by Adaltis, and the commercial name is MOLgen-COVID-19 Real Time RT PCR.

### Upcoming updates

The kit has recently been updated with detection of an additional gene, S (spike gene), which is read in the Cy5-5 channel. Figure 4 reports the curve and relative CT of a positive sample.

### Comparison test with other commercial kits

The comparison test was performed using 40 additional nasopharyngeal swabs that were examined with three commercially available kits (above reported) following the manufacturers’ instructions (Table 6).

All methods described were carried out in accordance with relevant guidelines and regulation and the study was approved by Independent Ethics Committee Tor Vergata Polyclinic on 25 June 2020.

### Ethics approval

The study did not include human participants but leftover samples. Specific informed consent are not required (as stated by “Independent Ethics Committee Tor Vergata Polyclinic on 17 June 2020, having based this study on the use of leftover human specimens collected for routine analysis that would otherwise been discarded. The same specimens are “unlinked anonymized materials”. This statement is in agreement to FDA “Guidance on Informed Consent for In Vitro Diagnostic Device Studies Using Leftover Human Specimens that are Not Individually Identifiable” April 25, 2006, and “Bioetica ed uso dei campioni biologici umani” Pezzati P. & Graziani MS. Biochimica Clinica, 2008, vol. 32, n. 3.

## Results

**Figure 1:**
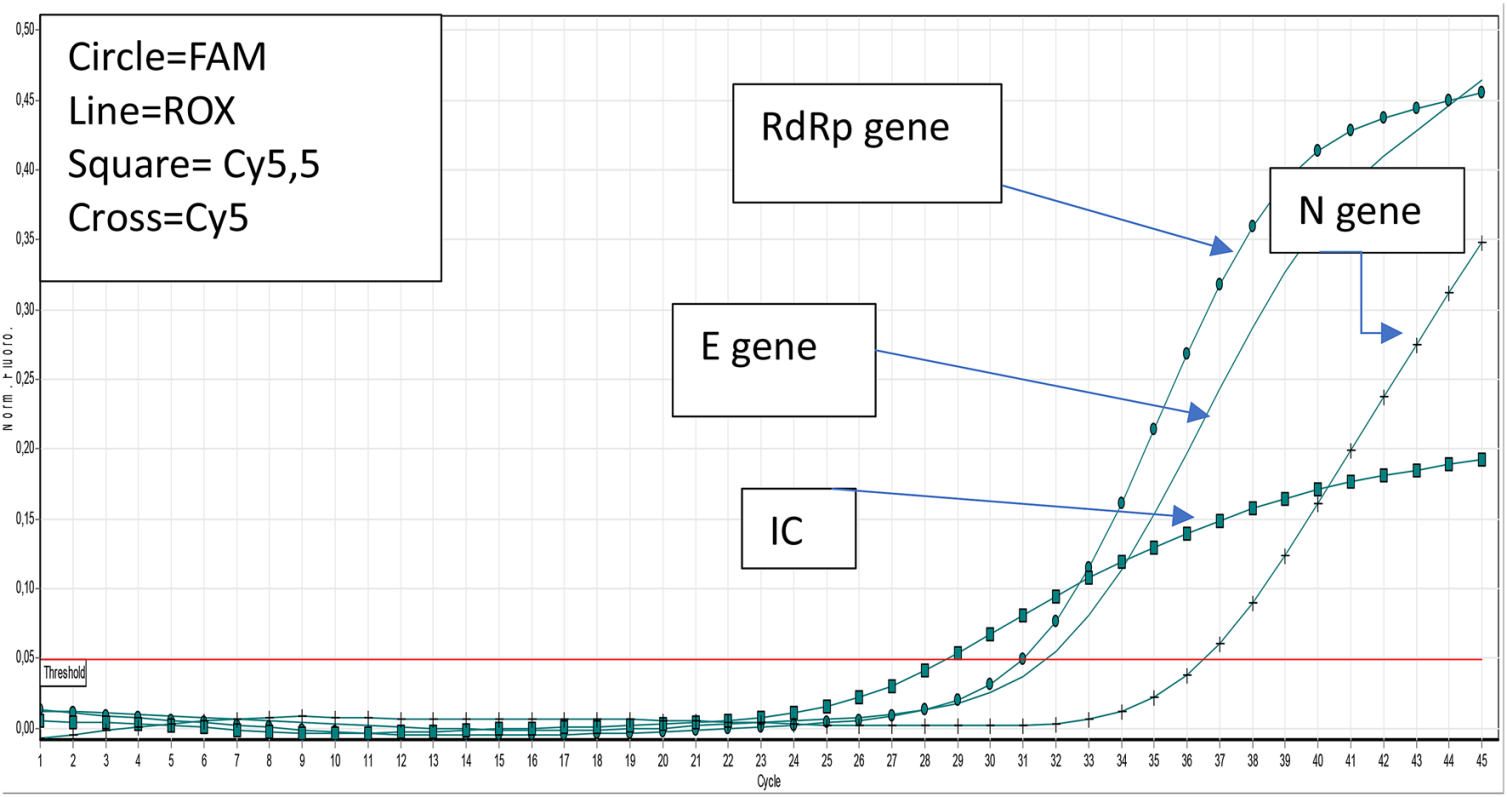
The figure illustrates the Real Time PCR curves of the genes detected by our kit; RdRp gene: RNA-dependent RNA polymerase gene; E: the envelope gene; N: the nucleocapsid gene; S: spike gene of COVID-19.

Positivity in our test was based on WHO guidelines^7^. In particular, a sample was considered positive if it showed a signal in at least one Rox (E gene) and/or Cy5 (N gene) fluorophore, while the presence of a single positive signal in the Fam channel was considered “inconclusive”, as the RdRp gene is common in other sarbecoviruses. In contrast, the absence of a signal in all channels, with the exclusion of Hex (that of β-actin), allowed us to conclude that a sample was negative. The absence of a signal in all channels identified a sample as “invalid” due to probable inhibition of the PCR or unreliable sampling. Table 1 reports the interpretation criteria. Figure 1 reports the curve and relative CT of a positive sample (Table 2).

**Table 1:** Specifity evaluation of our kit using NATtrol SARS-CoV-2 (E/ORF1ab recombinant), NATtrol SARS-CoV-2 and NATtrol Coronavirus SARS.

Organism	Results target RdRp	Results target E	Results target N
SARS-CoV-2 (recombinant)	Undetected	**Detected**	Undetected
SARS-CoV-2 (recombinant only N)	Undetected	Undetected	**Detected**
Coronavirus SARS	**Detected**	Undetected	Undetected

**Table 2:** Limit of Detection (LoD) of our assay evaluated using Research Reagent for SARS-CoV-2 RNA (NIBSC code 19/304) from the National Institute for Biological Standard and Control.

Sample no	RIDAGENE SARS-CoV-2 test (single target ORF1 ab gene)	Real-Time Fluorescent RT-PCR Kit for Detecting SARS-CoV-2-BGI (single target: E-gene)	MOLgen SARS CoV-2 Real Time RT PCR Kit (three targets, E, N, RdRp gene)	Allplex SARS-CoV-2 (four targets: E, N, RdRp/S gene)
34	Positive	Positive	Positive	Positive
1	Positive	Negative	Negative	Positive
1	Positive	Negative	Positive	Positive
1	Positive	Positive	Positive	Negative
1	Positive	Negative	Positive	Negative
1	Positive	Negative	Positive	Positive
1	Positive	Positive	Negative	Positive

**Figure 2:**
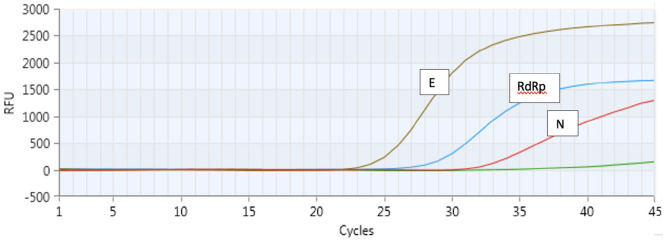
Figure illustrates the Real Time PCR curves of the genes detected by our kit; RdRp gene: RNA-dependent RNA polymerase gene; E: the envelope gene; N: the nucleocapsid gene of COVID-19. IC represents Internal Control (β-actin gene).

The results of our test showed the absence of any cross-reaction and evidence of a specific reaction of our primers and probes toward the COVID-19 genes; the data are shown in Tables 3 and 4 and Fig. 2. The LoD of our kit is shown in Table 5. The mean CT value for amplification of the β-actin gene in a negative sample was 27.81 ± 3.06.

**Table 3:** Results comparison of our assay (MOLgen SARS CoV-2) with three commercially available RT-PCR assays detecting SARS-CoV-2.

NIBSC	Dilution factor	Cp/mL	Cp/Reaction	Target RdRp	Target E	Target N
NIBSC code 19/304	1–1000	10,000	140	31.58	30.4	38.3
NIBSC code 19/305	1–2500	4000	56	32.9	31.4	–
NIBSC code 19/306	1 to 10,000	1000	14	34.78	32.63	–

**Table 4:** Interpretation criteria used in our qRT-PCR assay.

Target gene and channel	Example 1	Example 2	Example 3	Example 4	Example 5	Example 7
IC (β-actin) HEX	+/−	+/−	+/−	+/−	+	−
RdRp gene (FAM)	+	+	+	+	−	−
E gene (ROX)	−	+	+	−	−	−
N gene (Cy 5)	−	+	−	+	−	−
S gene (Cy 5.5)^a^	−	+	+/−	+/−	−	2
Results interpretation	Negative for CoVid19 Sarbecovirus detected	Positive for CoVid 19	Positive for CoVid 19	Positive for CoVid 19	Negative	Sample invalid probably inhibition or unsuitable withdrawal

Cut off value for all gene is ≤ 40; In presence of strong signal of others genes, the signal of IC may be inhibited but the results still valid.

In agreement with WHO guideline a sample is defined positive in presence of at least one specific gene among those detected (N, E, S); in our kit RdRp is in common with other sarbecovirus.

a = S gene has been recently added to our kit.

**Table 5:** Cross reactivity evaluation using NATtrol Respiratory Verification Panel (Catalog Number NATRV2-BIO) and NATtrol MERS-CoV Stock (Catalog Number NATMERS-ST).

Organism	Strain	Results (RdRp + E + N)
Influenza A	H1N1 A/New Caledonia/20/99	Undetected
Influenza A	H3 A/Brisbane/10/07	Undetected
Influenza A	2009 H1N1 A/NY/02/09	Undetected
Influenza B	B/Florida/02/06	Undetected
Metapneumovirus8**	Peru6-2003	Undetected
Respiratory Syn2ytial Virus A	N/A	Undetected
Rhinovirus 1A	N/A	Undetected
Parainfluenza virus Type 1	N/A	Undetected
Parainfluenza virus Type 2	N/A	Undetected
Parainfluenza virus Type 3	N/A	Undetected
Parainfluenza virus Type 4	N/A	Undetected
Adenovirus Type 3	N/A	Undetected
*M. pneumoniae*	M129	Undetected
*C. pneumonia*	CWL-029	Undetected
*C. pertussis*	A639	Undetected
Adenovirus Type 31	N/A	Undetected
Adenovirus Type 1	N/A	Undetected
*B. parapertussis*	A747	Undetected
Coronavirus NL63	N/A	Undetected
Coronavirus 229E	N/A	Undetected
Coronavirus OC43	N/A	Undetected
Coronavirus HKU-1	N/A	Undetected
MERS-CoV	Florida/USA-2Saudi Arabia_2014	Undetected

The diagnostic sensitivity and coincidence rate were evaluated by testing our kit with the Coronavirus SARS-CoV-NAT positive panel (catalog no. NPP-COV-001) supplied by Biomex (Biomex, GmbH Germany). The panel comprises 20 individual donor members containing coronavirus RNA inactivated in viral transport medium (VTM). Each test was conducted in triplicate after RNA extraction using our kit, and the coincidence rate was 0.96.

**Figure 3:**
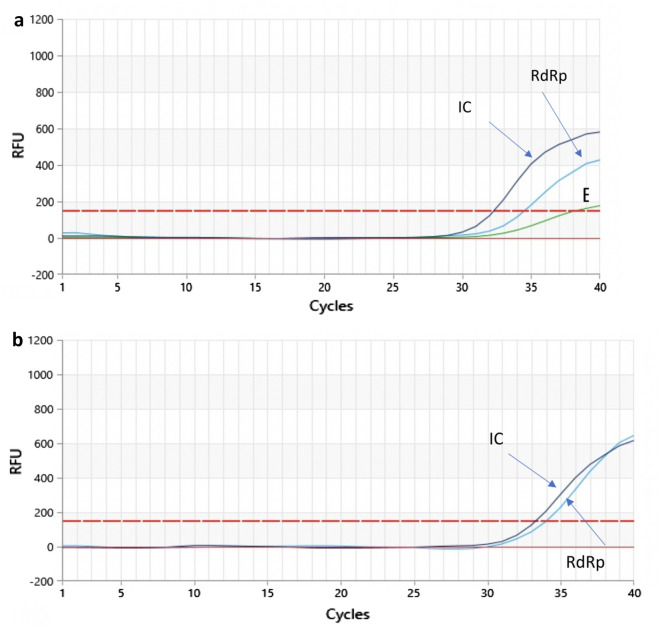
The figure illustrates the Results of qRT-PCR assays obtained using ZeptoMetrix panels. On y axis: Relative Fluorescence Unit (RFU); on x axis: threshold cycle (CT).

The study included 166 samples. A total of 133 samples were negative, 31 were positive, and two samples did not show amplification of the endogenous internal control (β-actin), which we concluded were indeterminate. Figure 3 shows examples of positive and inconclusive samples.

Of the 31 positive samples, 6 were only positive for RdRp and therefore reported as “inconclusive”, and 25 were positive for COVID-19. Among these 25, nine were positive for all objectives, while 16 were positive for RdRp and E only (see Table 2). The “inconclusive” samples were retested, and the results confirmed the detection of a single target (RdRp) that is designed to be common among sarbecoviruses. The CTs recorded in the Fam channel (RdRp) for these six samples were 33.86, 29.36, 36.52, 29.87, 36.51, and 35.28.

**Figure 4:**
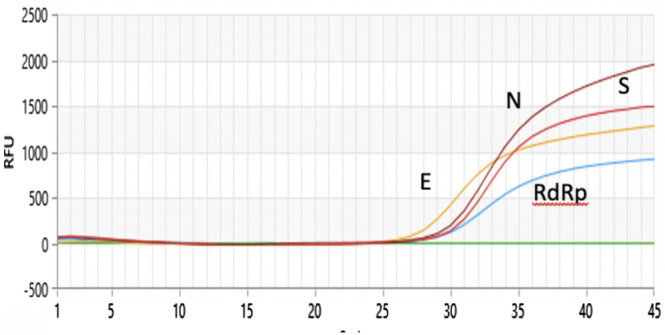
Box A shows a positive sample in which has been detected two target genes (RdRp and E). Box B shows a sample concluded as inconclusive being detected RdRp only.

The results shown by our assay agreed with those obtained by using Novel Coronavirus (Novel-Cov19) Multiplex Real-Time RT-PCR (RR-0479-02) (Liferiver Bio-Tech; US) for the initial diagnosis of SARS-CoV-2 (Fig. 4).

Finally, Table 6 reports the results of the comparison test. Only two samples showed discrepant results by our assay. In one case, the sample could be considered “inconclusive”, with two positive and two negative findings. In the second case, our assay gave a negative result compared to a positive result obtained with other methods. This sample was concluded as a false negative. However, the global performance of our assay was good, with the results of 38/40 samples (95%) in agreement with those recorded for at least two different assays^18^.

**Table 6:** Results obtained by our assay (qRT-PCR).

Results	Target gene				No. sample
	RdRp	N	E	β-actin	
Indeterminate	–	–	–	–	2
Inconclusive	6			6	6
Positive	9	9	9	9	9
	16		16	16	16
Negative				133	133
Total					166

S gene was not present in our kit at the time of the initial evaluation here reported.

## Discussion

Accurate and reliable diagnostic analysis and large-scale testing are essential for the early detection of pathogens related to disease outbreaks, but it is even more important to take timely actions for the public health during pandemic events. This has proven to be true for SARS-CoV-2, which was identified as the cause of an outbreak of pneumonia in Wuhan, China, in December 2019 and rapidly spread around the world^19–26^. Laboratory diagnosis of COVID-19 infections caused by severe acute respiratory syndrome coronavirus 2 (SARS-CoV-2) is achieved primarily by performing nucleic acid amplification tests (NAATs) on samples from the respiratory tract. Indeed, upper respiratory tract specimens, such as nasopharyngeal swabs and oropharyngeal swabs, generally have elevated SARS-CoV-2 viral loads at the onset of symptoms^26^. Some authors have recently suggested expanding NAATs to include saliva and stool samples, but the debate is still ongoing^27,28^. Due to the urgent worldwide request for tests to diagnose COVID-19, many NAATs for SARS-CoV-2 are available, and many others are in the final stages of development^25,29–31^. This development has the great advantage of making a wide range of diagnostic tests available to health systems and allows health care providers to respond to the diagnostic needs caused by the pandemic. On the other hand, the staggering number of kits available around the world coupled with the differences between the NAATs pose problems in the validation process. An important limit in qRT-PCR validation assays that detect SARS-CoV-2 is the availability of standard RNA viruses. Additionally, it is the subject of an ongoing discussion whether to consider the full-length integral SARS-CoV-2 RNA as a safety level 3 biohazard^24,25^. If the debate reaches a consensus to mark SARS-CoV-2 as Level 3, its treatments could only be performed by laboratories with adequate level 3 (BSL3) security measures, but this limits the ability to perform experimental tests^29^. With these critical issues in mind, we have developed a qRT-PCR assay capable of detecting three SARS-CoV-2 target genes. The strongest aspect of our assay resides in the IC, with β-actin being a conserved gene present in all human cells, and its detection is useful to establish the reliability of the sampling. In fact, the accurate collection of nasopharyngeal samples has revealed a crucial aspect of the preanalytical phase that strongly conditions the results of the NAAT, being one of the most frequent and probable causes of false-negative results and therefore of a late diagnosis^19,29,31^. Additionally, to avoid working with full-length viral RNA and to overcome the problem of BSL3, we chose to build an artificial chimeric plasmid to test our primers and probes while using ZeptoMetrix panels that allowed us to evaluate the specificity of our assay under safe conditions. Additionally, the performances of our assay were very good in comparison to three commercially available kits. Only two discrepant results, an “inconclusive result” and a possible “false negative”, were observed. On the other hand, differences in the detection of SARS-CoV-2 are well known and described in the literature, and they are the result of a variety of factors, including the target gene and the CT threshold chosen to define a positive sample (some methods go beyond 39 CT, which means a very low viral load)^18,32^.

Another powerful aspect of our kit is that it is intended to be open, either for the nucleic acid extraction step or in the RT-PCR assay that will be performed on several instruments (in this paper, we tested two of them). Therefore, our assay can be used in any molecular biology laboratory. Additionally, our kit is free from the industrial production logic of “closed systems” and, conversely, is hypothetically available for distribution in large quantities. This aspect, at a time of great demand for tests and the well-known shortcomings of commercial kits, can be a significant strength in facilitating introduction into microbiological laboratories^33^.

Moreover, we have considered the potential genetic drift of SARS-CoV-2, especially as the virus evolves within new populations. Although the literature suggests that at least two specific molecular targets should be included in an assay to reduce the probability of cross-reactions, we have added a fourth target gene codifying the glycoprotein spike (S) gene, but validation tests are still in progress^29,30,34,35^.

Finally, as evidenced in the literature, in addition to direct respiratory sampling, rectal swabs and saliva may be suitable specimens to enhance the diagnosis of COVID-19, and we are expanding our assay validation test in this direction^28,29^.

## Data Availability

All data are provided in full in the results section of this paper. This paper was preprint from MedRxiv https://www.medrxiv.org/content/10.1101/2020.06.17.20124396v1.

## Abbreviations

qRT-PCR: Reverse transcriptase real time polymerase chain reaction
RdRp: RNA-dependent RNA-polymerese
E: Envelope
N: Nucleocapside
S: Spike
LoD: Limit of detection
WHO: World Health Organization
CDC: Center for Disease Control
ECDC: European Center for Disease Control
FDA: Food and Drug Administration
IC: Internal control
CP: Chimeric plasmid
VTM: Viral transport medium
